# ASET: an end-to-end pipeline for quantification and visualization of allele specific expression

**DOI:** 10.1186/s12859-025-06282-2

**Published:** 2025-10-21

**Authors:** Weisheng Wu, Kerby Shedden, Claudius Vincenz, Chris Gates, Beverly Strassmann

**Affiliations:** 1 BRCF Bioinformatics Core, University of Michigan, Ann Arbor, MI 48109 USA; 2 Department of Statistics, University of Michigan, Ann Arbor, MI 48109 USA; 3 Research Center for Group Dynamics, Institute for Social Research, University of Michigan, Ann Arbor, MI 48106 USA; 4 Department of Anthropology, University of Michigan, Ann Arbor, MI 48109 USA

**Keywords:** Allele-specific-expression (ASE), RNA-Seq, Nextflow, Parent-of-origin (PofO) effect

## Abstract

Allele-specific expression (ASE) analyses from RNA-Seq data provide quantitative insights into genomic imprinting and the genetic variants that affect transcription. Robust ASE analysis requires the integration of multiple computational steps, including read alignment, read counting, data visualization, and statistical testing—this complexity creates challenges for reproducibility, scalability, and ease of use. Here, we present ASE Toolkit (ASET), an end-to-end pipeline that streamlines SNP-level ASE data generation, visualization, and testing for parent-of-origin (PofO) effect. ASET includes a modular pipeline built with Nextflow for ASE quantification from short-read transcriptome sequencing reads, an R library for data visualization, and a Julia script for PofO testing. ASET performs comprehensive read quality control, SNP-tolerant alignment to reference genomes, read counting with allele and strand resolution, annotation with genes and exons, and estimation of contamination. In sum, ASET provides a complete and easy-to-use solution for molecular and biomedical scientists to identify and interpret patterns of ASE from RNA-Seq data.

**Supplementary Information:**

The online version contains supplementary material available at 10.1186/s12859-025-06282-2.

## Introduction

Allele-specific expression (ASE) is measurable when the two alleles are distinguishable at heterozygous single nucleotide polymorphism (SNP) sites. Unbalanced ASE can arise from multiple biological mechanisms, including genomic imprinting [1], regulatory genetic variation and eQTLs [2, 3], allele specific methylation or chromatin remodeling [4], X chromosome inactivation [5], and nonsense-mediated decay [6]. High-throughput RNA-Seq technology has been widely used to measure ASE. Multiple approaches and algorithms have been developed for ASE quantification, focusing on reducing the alignment bias towards reference alleles because the genome reference does not contain the alternative alleles [7]. AlleleSeq [8] and SNPsplit [9] can incorporate the alleles of the phased variants into the reference to create two haploid sets of genomes. After alignment against this personalized genome, the reads can be filtered to keep only the reads that are uniquely assigned to one of the haploid genomes. However, this approach requires complete phasing of the variants, which in most cases can only be achieved by sequencing the parental genomes. GSNAP [10] is a SNP-tolerant aligner that treats alternative alleles as matches to the reference, rather than counting them as mismatches, thereby reducing alignment bias toward the reference allele. WASP [11] is an alignment filtering method that swaps the alleles in SNP-containing reads, and then the reads whose mapping locations change after allele swapping can be eliminated. WASP is integrated into STAR [12, 13] which is a frequently used aligner for RNA-Seq reads due to its accuracy and speed. ASEReadCounter is a tool in the widely used GATK toolkit [14] and is specifically designed for allele-specific RNA-Seq read counting, with many available parameters controlling read filtering and counting criteria. ASElux [15] is an ultra-fast allele-specific read counter that first generates SNP-aware genome indices using only SNP-containing genic regions and then aligns the reads only against these regions for read counting. Allelome.PRO [16] is a pipeline for identifying ASE from user-provided RNA-Seq alignments and phased SNP data. It was originally tailored for mouse reciprocal cross samples and was later expanded to diverse biological samples including human datasets. Most of the tools mentioned above have been reviewed, benchmarked, and widely adopted for ASE analyses [17], and the STAR-WASP-ASEReadCounter workflow was used to generate SNP-level ASE data in the Genotype-Tissue Expression (GTEx) project [18, 19].

Pipelines have been developed to incorporate some of these tools for ASE quantification, such as the gtex-pipeline [18], mRNAseq from snakePipes [20], Allele-specific RNA-seq workflow (https://github.com/yuviaapr/allele-specific_RNA-seq), RNAseq-VAX (https://github.com/arontommi/RNAseq-VAX), and as_analysis (https://github.com/aryarm/as_analysis. However, most of these pipelines lack either flexibility or end-to-end analyses; notably, none of these pipelines directly include ASE data visualization or PofO testing.

Here we present ASE Toolkit (ASET) for SNP-level ASE quantification. ASET leverages the Nextflow workflow manager [21] that accepts raw short-read RNA-Seq data and produces SNP-level ASE count data with gene annotation and contamination estimates. ASET integrates multiple alignment options that were designed specifically for ASE analysis, enabling simple usage and customization. It also includes data visualization and PofO testing. ASET provides an easy-to-use suite that streamlines ASE data preparation and visualization, providing the foundation for further interpretation and analysis.

## Methods

### Overview

The main modules of ASET are implemented using Nextflow, a modern workflow management system that enables scalable, reproducible, and portable computational pipelines. Nextflow is widely used in the bioinformatics community due to its comprehensive documentation, container support, and mature community on GitHub and Slack. Leveraging the latest DSL2 syntax, ASET adopts a modular design in which individual analysis steps are implemented as modules. This modularity allows for clean organization, simplified maintenance, and the seamless integration of sub-workflows for alternative analysis paths. ASET also supports containerization through Docker [22] and Singularity [23], enabling portable execution across local machines, HPC clusters, and cloud environments. Reproducibility is further enhanced by version-controlled releases, locked software dependencies via containers, and automatic reporting of tool versions and parameters. Analysis parameters and computational parameters (e.g. CPU and memory usage) can be specified via a configuration file.

The data visualization functionality is bundled in an R [24] library “ASEplot”. R is a very common platform used for data analysis and visualization. The PofO testing algorithm is provided as a Julia [25] script. Julia is a high-performance programming language designed for statistical modeling.

**Fig. 1:**
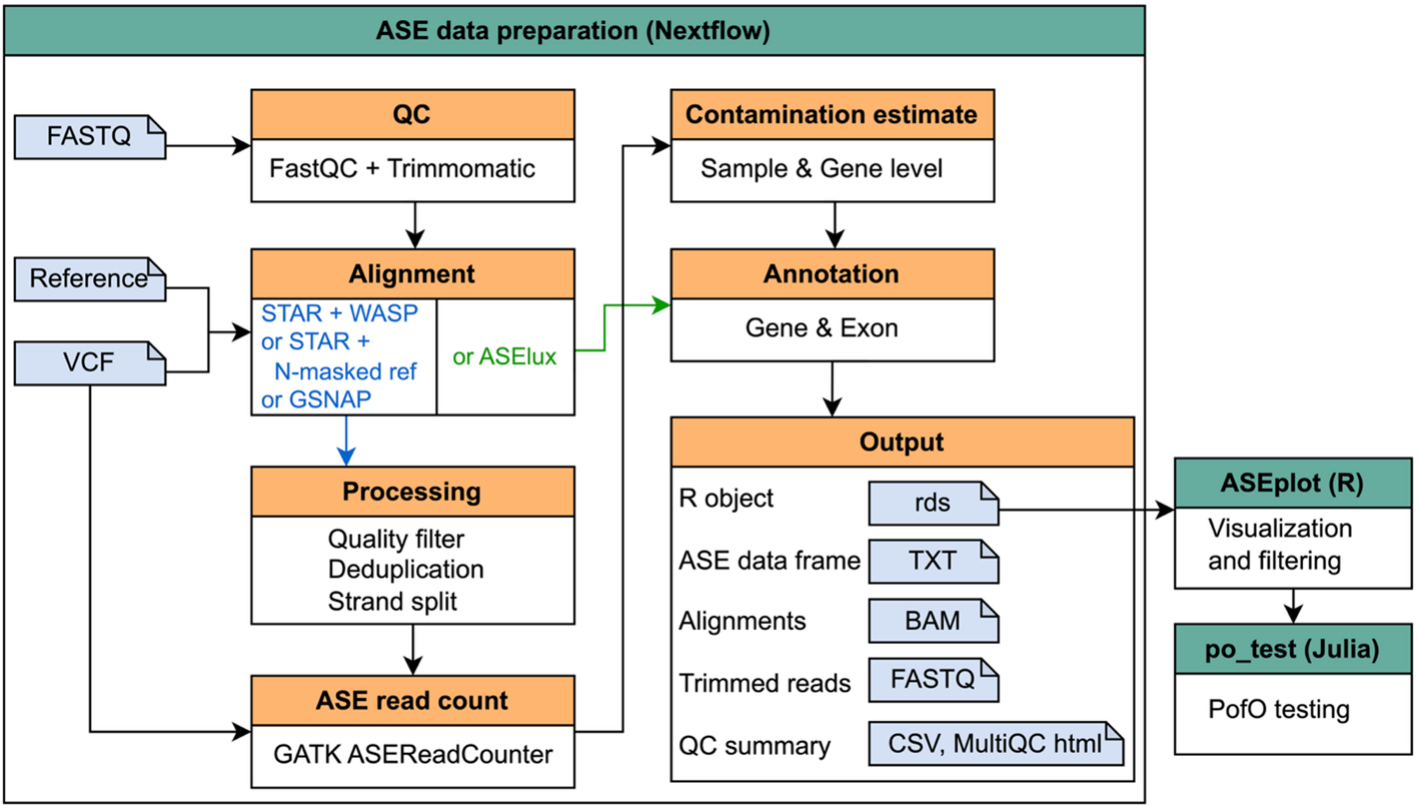
Overview of ASET. This flow chart illustrates the main steps of ASET, including a Nextflow-based workflow for preparing ASE count data, an R-based component for data visualization and filtering, and a Julia-based component for parent-of-origin (PofO) testing

An overview of the ASET pipeline is shown in Fig. 1. It requires two input files: a sample sheet containing the paths to the read files and SNP VCFs, and a parameter configuration file for adjusting parameter settings for each tool and the paths to reference files. ASET can be run in two modes: *from_fastq* or *from_bam*. In the *from_fastq* mode, it takes the raw FASTQ reads as input and implements read QC, trimming, and alignment. In the *from_bam* mode, it takes the provided BAM files and goes directly to alignment filtering and deduplication. Users also need to provide a VCF containing the SNPs for each sample and this VCF will be used for SNP-aware alignment and SNP-level ASE read counting. After read alignment and counting, the data will be concatenated from all the samples to produce an ASE data table, followed by contamination estimation and annotation for genes and exons. The output can be loaded directly into ASEplot for plot generation and data filtering. ASET does not require phasing of the SNPs, but when phased SNPs are available, phasing information can be incorporated, and the phased subset can be analyzed using po_test.jl for PofO testing.

The comparison of capabilities among ASET and other available ASE pipelines is summarized in Table 1. The advantages of ASET include: (1) incorporation of four commonly used alignment approaches tailored for ASE analysis, (2) generation of ASE count data in a strand-specific manner, (3) estimation of contamination levels, (4) data visualization, and (5) PofO testing.

**Table 1:** Comparison between ASET versus other available ASE pipelines. (NA means “not directly available”)

Feature	ASET	gtex-pipeline	snakePipes	Allele-specific RNA-seq workflow	RNAseq-VAX	as_analysis
System	Nextflow	Cromwell	Snakemake	Nextflow	Nextflow	Snakemake
Aligner	GSNAP or		STAR or HISAT2 with N-masked ref	STAR with N-masked ref	NA	STAR + WASP
	STAR + WASP or	STAR + WASP				
	STAR with N-masked ref or ASElux					
Strand-specific	Supported	NA	Supported	NA	NA	NA
Read counting	SNP-level^#^	SNP & Haplotype-level^# ϕ^	Gene-level	Gene-level	SNP-level^#^	SNP-level
Contamination estimate	Supported	NA	NA	NA	NA	NA
Visualization plots	Tailored for ASE	NA	Tailored for QC and differential expression	NA	NA	NA
PofO testing	Supported	NA*	NA	NA	NA	NA

^#^These pipelines use GATK ASEReadCounter for SNP-level allelic read counting from alignments

^ϕ^The gtex-pipeline has a module for haplotype-specific expression when phased genotypes are available

*The gtex-pipeline has a module for eQTL testing

### Detailed pipeline steps

#### Read QC

ASE data accuracy and robustness depend heavily on the quality of sequencing data, especially the effective coverage of the assayed SNPs, as shown in our previous publication [26]. ASET uses FastQC [27] and CollectRnaSeqMetrics from GATK [14] to assess RNA-Seq read quality, and uses Trimmomatic [28] to remove adapter contamination and low-quality ends. QC metrics are summarized in both a MultiQC [29] report and a tabular spreadsheet.

#### Read alignment

ASET currently contains four sub-workflow choices for read alignment. The mapper parameter specified in the configuration file selects one of these alignment approaches: (1) STAR + WASP where the alignment is performed using STAR with the –waspOutputMode parameter to enable WASP filtering; (2) STAR + NMASK where the genome is first N-masked at the SNP sites and then used for STAR alignment; (3) GSNAP where reads are aligned using GSNAP in the SNP-tolerant mode; and (4) ASElux where reads are aligned and counted using ASElux. When using ASElux, raw reads instead of trimmed reads will be used, as ASElux generates errors with trimmed reads, likely due to variable read lengths. Note that the provided genome FASTA and GTF files will be indexed by the chosen aligner for splice-aware alignment.

#### Alignment filtering, deduplication, and strand separation

Alignments are filtered based on adjustable flags and mapping quality cutoffs. STAR + WASP-based alignments can additionally exclude alignments flagged as problematic (based on vW tag). Reads are then deduplicated using GATK MarkDuplicates. Deduplicated reads are split into two alignment files based on strand. A strandedness parameter needs to be provided to indicate whether read 1 or read 2 corresponds to the original RNA strand. Note that ASElux-based alignments skip this step as ASElux integrates both read alignment and counting without outputting the alignment files for manipulation.

#### ASE read counting

GATK ASEReadCounter is applied on each alignment file to compute allele-specific read counts on all provided heterozygous and homozygous SNPs and optionally also for the genotyped reference sites. Output files on different strands from all samples are concatenated into a single file for each type of site. Base quality cutoffs, mapping quality cutoffs, and the overlap handling scheme are configurable. As above, ASElux-based alignments skip this step.

While the STAR_WASP alignment routine combined with read counting by ASEReadCounter is based on the GTEx workflow, we enhanced it by adding the capability to split read counts by strand (Supplementary Fig. 1).

#### Contamination estimation

The average non-alternative-allele frequency on homozygous SNP sites and the average non-reference-allele frequency on reference sites (if available) are calculated to serve as an estimate of cross-contamination (or mislabeling) for each sample. For placental samples where maternal contamination is a concern, the average non-reference-allele frequency at the reference sites where the mother has a non-reference genotype is also calculated for each gene individually, with the assumption that the non-reference allele counts arise from contamination by maternal tissue. ASElux-based alignments skip this step since ASElux only counts reads at exonic heterozygous SNPs.

#### Annotation

Based on the provided GTF, the exons from the same gene are merged into a union exon set and then used to annotate a table of SNPs. Each SNP (row) details exon coordinates, gene IDs, symbols, and gene types. When phasing data is provided, paternal and maternal alleles will be indicated, and the paternal allele frequency will be calculated for each SNP that has data.

#### ASET outputs

ASET generates allele-specific read count data at user-specified heterozygous SNPs, integrating gene and exon annotations, contamination estimates, and phasing information if available. Outputs include both human-readable tabular files and a consolidated RDS object containing (1) the ASE count table and (2) merged union exons for each gene. The pipeline additionally produces trimmed FASTQ files, alignment BAM files, MultiQC reports, and a comprehensive QC tabular spreadsheet.

#### Data visualization with ASEplot

This RDS file produced by ASET can be loaded into R, where the ASEplot library offers convenient functions for data visualization, such as displaying SNP positions relative to genes and plotting ASE distributions across samples at both the gene and SNP levels.

#### Determination of parent-of-origin scores

To quantify the allelic bias that is due to imprinting and associated with parent-of-origin (PofO) from bias that is caused by sequence variants, we developed a method that distinguished between these two potential causes for ASE. PofO ASE arises from differential imprinting between paternal and maternal alleles, resulting in an association between ASE and parental origin across individuals. In contrast, genetic ASE is typically driven by cis-acting genetic variants, producing an association between ASE and specific SNP alleles across individuals. We developed a statistical method, as described below, to jointly model these two types of effects, enabling the identification of PofO ASE events.

For a given gene with *N* total read counts and *m* distinct SNPs, let *Y*_*ijk*_ denote the read count for allele *k* of SNP *j* for subject *i*. The alleles are coded *k* = 0, 1 for the reference and alternative alleles, respectively. Define *X*_*ijk*_ = 1/2 when *k* = 0 and − 1/2 when *k* = 1; and define *Z*_*ijk*_ = 1/2 and − 1/2 for paternal and maternal allele read counts, respectively. Next, construct an *N* × *m* matrix of indicator variables *U*, where column *l* of *U* is defined as *U*^*l*^_*ijk*_ = 1 if *j* = *l* and 0 otherwise. Next, let *V* denote an *N* × *q* matrix consisting of the left singular vectors of *U* whose singular values are at least 1% of the maximum singular value of *U*. We fit a cluster-robust quasi-Poisson regression model for each gene in which the indices *i*, *j*, *k* index the *N* observations, and the explanatory variables are the main effect of parent of origin (*Z*_*ijk*_), the main effect of ref/alt status (*X*_*ijk*_), main effects for SNP indicators (*V*), and all pairwise interactions between SNP indicators (*V*) and ref/alt status (*X*_*ijk*_). Including the *X* and *V* main effects and their pairwise interactions allows us to account for genetic ASE, while clustering on subjects (*i*) allows us to account for correlations among read counts within the same individual (e.g. due to linkage disequilibrium). The full model is shown below:

$$\begin{aligned} {\text{log}}\left( {E\left[ {Y_{{\left\{ {ijk} \right\}}} } \right]} \right) = & \beta _{0} + po*Z_{{\left\{ {ijk} \right\}}} + \beta _{1} X_{{\left\{ {ijk} \right\}}} \\ & + sum_{{\left\{ {l = 1} \right\}}}^{q} \gamma _{l} V_{{\left\{ {ijk} \right\}}}^{{\left\{ l \right\}}} + sum_{{\left\{ {l = 1} \right\}}}^{q} \delta _{l} \left( {V_{{\left\{ {ijk} \right\}}}^{{\left\{ l \right\}}} *X_{{\left\{ {ijk} \right\}}} } \right) \\ \end{aligned}$$

We refer to the estimated coefficient for *Z* as the PofO score and denote it *po*, with its z-score denoted *po_z*. Positive and negative *po* correspond, respectively, to paternally and maternally biased expressions, while 0 denotes a balance. We view |*po*|> 3 as denoting strong parentally determined ASE, implying at least a 20-fold difference between the two alleles, and |*po_z*|> 3 as denoting statistical significance.

## Results

### Execution statistics

We tested the four routines of ASET with a set of ten 150 bp Illumina PE targeted RNA-Seq samples whose read pair counts ranged between 26 and 107 million, with the average being 66 million. The execution statistics are shown in Table 2. As expected, the GSNAP routine took the longest time because of the slowness of read alignment by GSNAP. The ASElux routine was ultra-fast since ASElux only aligns the SNP-containing reads.

**Table 2:** Execution statistics of the four routines of ASET. Comparison of computational performance across four routines implemented in ASET (STAR_WASP, STAR_NMASK, GSNAP, and ASElux). For each routine, the number of executed tasks, total runtime duration (in hours), cumulative CPU usage (in CPU-hours), and peak memory consumption (in gigabytes) are reported

	STAR_WASP	STAR_NMASK	GSNAP	ASElux
Tasks	132	151	153	61
Duration (h)	8.2	10.9	150.3	2.5
CPU-Hours (h)	466.7	491.2	6674.8	10.1
PeakMemory (G)	48.6	49.7	33.9	34.5

### Visualization generated with ASEplot

**Fig. 2:**
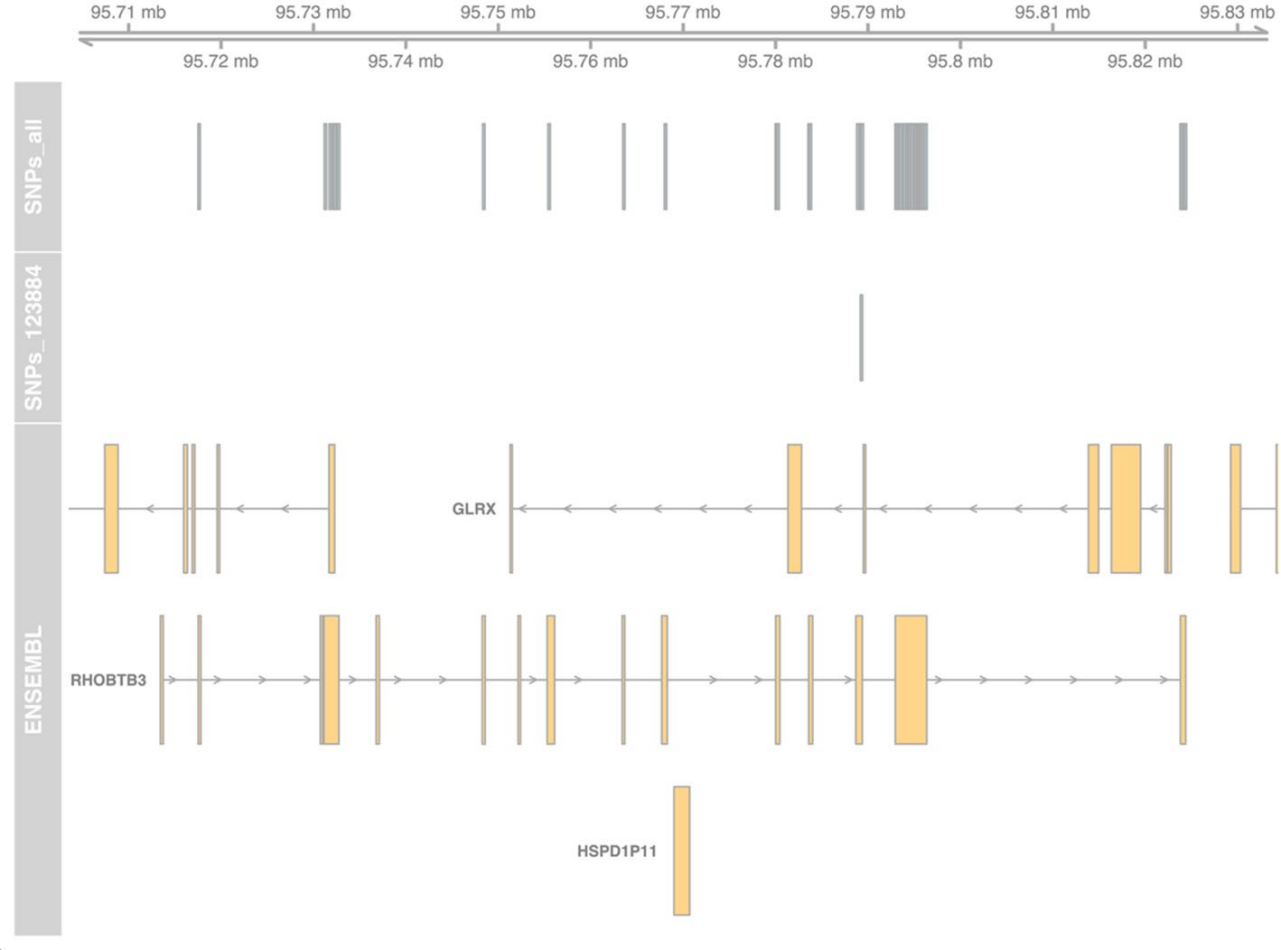
SNP locations in the RHOBTB3 gene locus, with isoforms collapsed into a single model per gene. The “SNPs_all” track shows all assayed heterozygous SNPs in this gene; the “SNPs_123884” track shows only the SNPs detected in the specified sample; and the “ENSEMBL” track displays the gene models

**Fig. 3:**
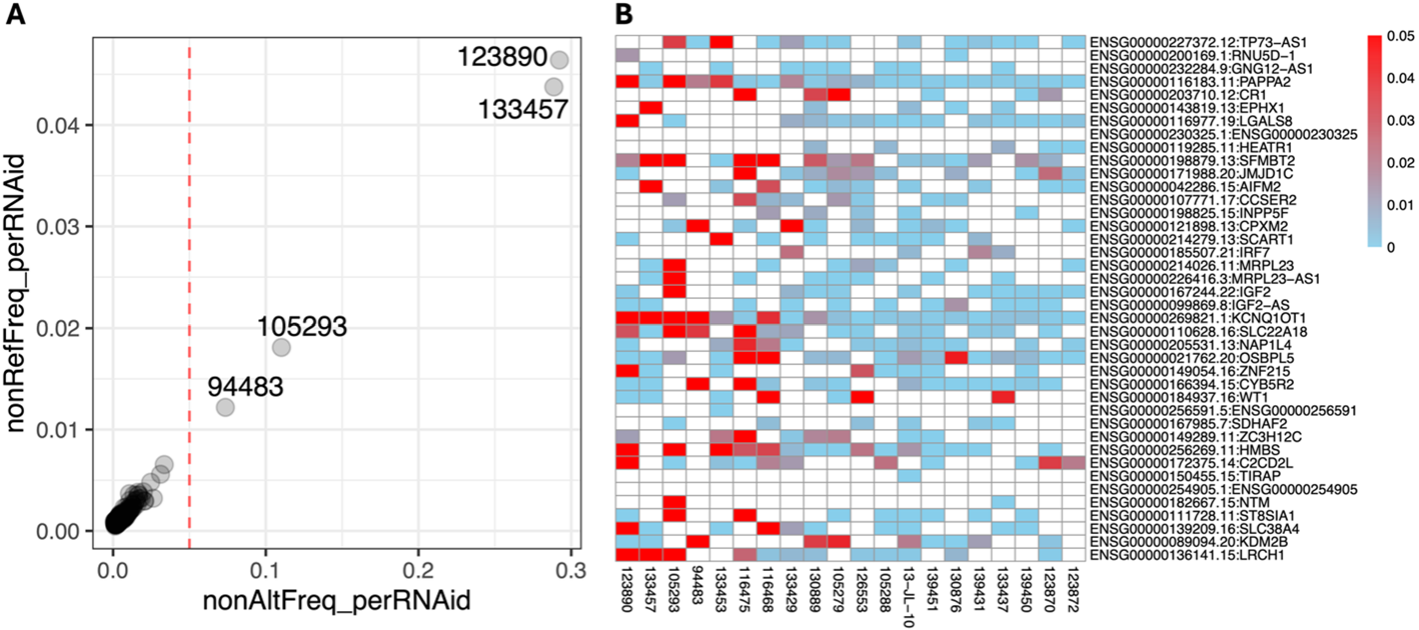
Contamination estimated from opposite allele frequencies at homozygous sites. **A** Scatter plot of contamination estimates averaged per sample. The dotted vertical line at 5% indicates a user-defined cutoff. Both non-reference allele frequency at reference sites and non-alternate allele frequency at homozygous SNP sites are shown. **B** Heatmap of contamination estimates averaged per gene, based on non-reference allele frequency at reference sites. Only the data from a subset of genes in a subset of samples are displayed

**Fig. 4:**
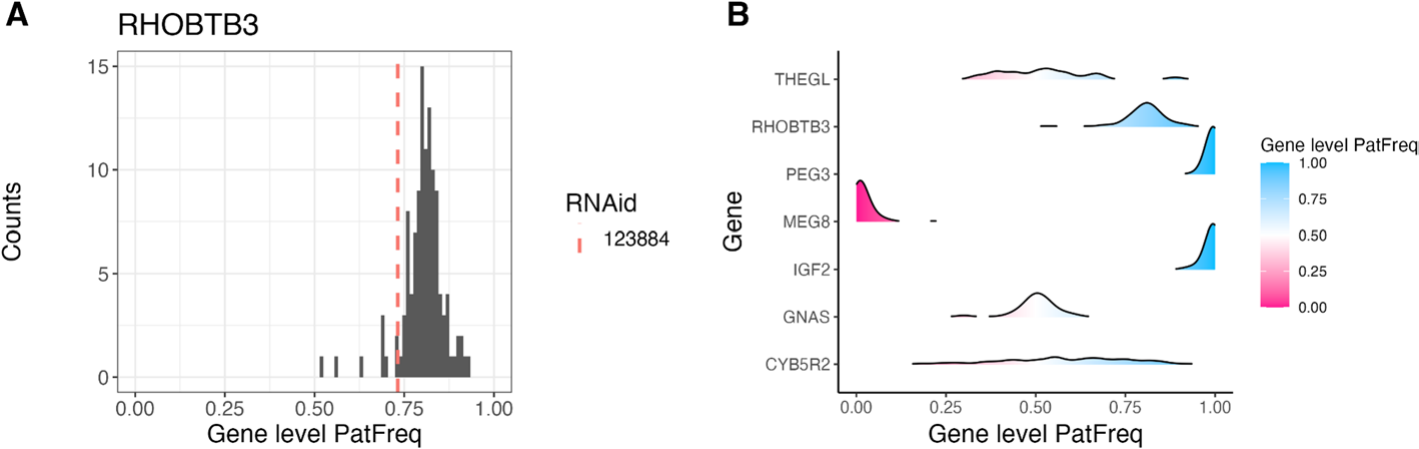
Distribution of gene-level paternal allele frequency, shown as **A** a histogram for one gene with the sample of interest marked; or **B** ridges for multiple genes, with color indicating a tendency for paternal (blue) or maternal (pink) specific expression. Gene-level paternal allele frequency was calculated by summing paternal and total count data from the exonic SNPs and then taking the ratio

**Fig. 5:**
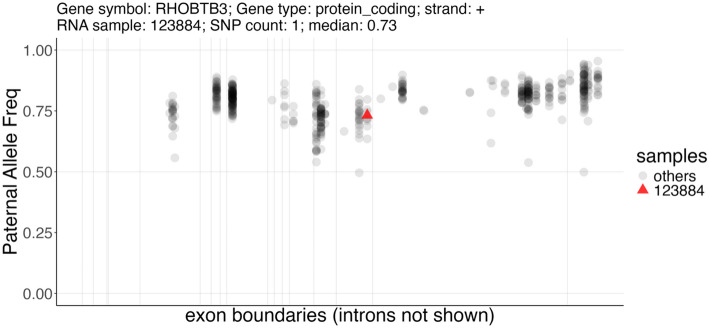
Distribution of SNP-level paternal allele frequency across different samples in a gene, shown as a scatter plot where vertical lines represent exon boundaries after merging for each gene. When a sample ID is specified, it is marked as a red triangle where all other samples are shown as gray round dots. The SNP count and the median allele frequency for this sample, plus the gene information, are shown in the title

We applied ASET on the sequencing data from a set of 244 targeted RNA-Seq samples we previously published [26], using the STAR + WASP alignment approach. This produced a data table with 346,503 exonic SNP × sample × strand data points, observed in 783 genes. Using the ASEplot R library, we visualized the SNP locations in specific genes (Fig. 2 and Supplementary Fig. 2), sample-level and gene-level contamination (Fig. 3), and exon- and gene-level ASE distribution across different samples, exons, or genes (Figs. 4, 5, and Supplementary Fig. 3). After data filtering including requiring at least 10 read counts at SNPs and lower than 5% contamination (when measurable), 264,046 data points were retained. The phased subset with 125,772 data points was analyzed using po_test.jl for PofO testing. The results showed that out of 392 genes that were testable, 153 had a strong PofO effect with |*po_z*|> 3, with 92 biased to paternal expression and 61 biased to the maternal side. Among these genes, 33 had a large difference between the alleles with |*po*|> 3.

### Genes with parent-of-origin effect

We applied our PofO testing method to the phased subset of ASE data (“Visualization generated with ASEplot” Section) and identified 154 genes with significant PofO effects, using a |*po_z*|> 3 cutoff. Comparison with a previously reported placenta-specific imprinted gene set [30] demonstrated strong concordance (Supplementary Table 1).

## Discussion

ASET provides an integrated and reproducible framework for the generation and visualization of ASE data, addressing a critical need for streamlined ASE analysis in transcriptomics studies. It combines a robust Nextflow-based workflow for data preprocessing with a dedicated R package for visualization and a statistical algorithm for PofO testing. Compared to other available ASE workflows, ASET provides a more complete solution by including multiple alignment approaches tailored for ASE analysis, support for strand-specific read counting, contamination estimation, data visualization, and PofO testing. ASET employs containerization through Docker and Singularity to boost convenience and reproducibility across different environments.

The pipeline's modular structure provides flexibility for further expansion by the addition of more modules. For example, another sub-workflow can be added to enable personalized diploid genome construction and alignment when a complete phased SNP set is available. The current annotation of the SNPs by using the merged exons lacks the ability to interrogate isoform-level ASE. With diploid genome construction and sufficient density of heterozygous SNPs (e.g. from inbred mouse strains), there are approaches to resolve ASE quantification on the isoform-level [31, 32]. However, the best solution for isoform ASE analysis may lie in full-length transcriptome sequencing using long-read sequencing technologies [33, 34]. The current support provided for downstream data analysis focuses on basic visualization and PofO testing. We realize that there are a variety of methods for downstream analyses, such as eQTL and prediction of *cis*-acting ncRNA-targets [35]. In addition, haplotype-specific expression can be enabled using phASER, especially when long-read RNA-Seq data are available [36]. We will be working on adding more functionality to ASET to incorporate diploid alignment, isoform-level ASE measurement, and further statistical analysis, especially when phenotype data are available.

Overall, compared to the existing alternative pipelines, ASET provides a more comprehensive workflow that bridges the gap between raw data and SNP-level ASE measurement and interpretation, and is particularly valuable for studies of such phenomena as genomic imprinting, eQTLs, X chromosome inactivation and nonsense-mediated decay, where the preparation of robust ASE data is required.

## Supplementary Information

Below is the link to the electronic supplementary material.

Supplementary Material 1.

## Data Availability

ASET is available at https://github.com/weishwu/ASET. The ASE data preparation section is implemented in Nextflow with DSL2 syntax. The data visualization functionality is provided through an accompanying R package, ASEplot, available from GitHub (https://github.com/weishwu/ASEplot) or Docker Hub (https://hub.docker.com/r/weishwu/aseplot). The parent-of-origin (PofO) testing algorithm is implemented in a Julia script distributed with ASEplot. The RNA-Seq FASTQ files and the genotype data used to test the pipeline were published in our previous paper [26], and deposited in dbGaP as phs001782.v2.

## Abbreviations

ASE: Allele-specific expression
SNP: Single-nucleotide polymorphism
PofO: Parent-of-origin
