# Supplementary material for: ASET: an end-to-end pipeline for quantification and visualization of allele specific expression
Source: BMC Bioinformatics. 2025 Oct 21;26:257. doi: 10.1186/s12859-025-06282-2 (PMC12539063; doi:10.1186/s12859-025-06282-2)
Supplement: Supplementary file 1 — Supplementary Material 1. [file 12859_2025_6282_MOESM1_ESM.docx]

Supplementary materials for “**ASET: An end-to-end pipeline for quantification and visualization of allele specific expression”**

Supplementary Figure 1. Comparison of ASE read counts between ASET (STAR+WASP alignment approach) and GTEx. Sequencing reads from a sample were analyzed using both ASE with the STAR_WASP routine and the GTEx read alignment, deduplication and ASE read counting. GTEx analysis was performed according to <https://github.com/broadinstitute/gtex-pipeline/blob/master/rnaseq/README.md>. Since GTEx workflow does not separate strands while ASET does, the stranded read counts from ASET were summed before comparing with the counts from GTEx. The scatter plot in (A) shows that the correlation is very strong on both reference and alternate alleles. The remaining differences could be caused by different parameter settings, filtering cutoffs, and software versions. Based on the ASET output, about 40% of the SNPs have read counts on both strands, as shown in the pie chart (B).


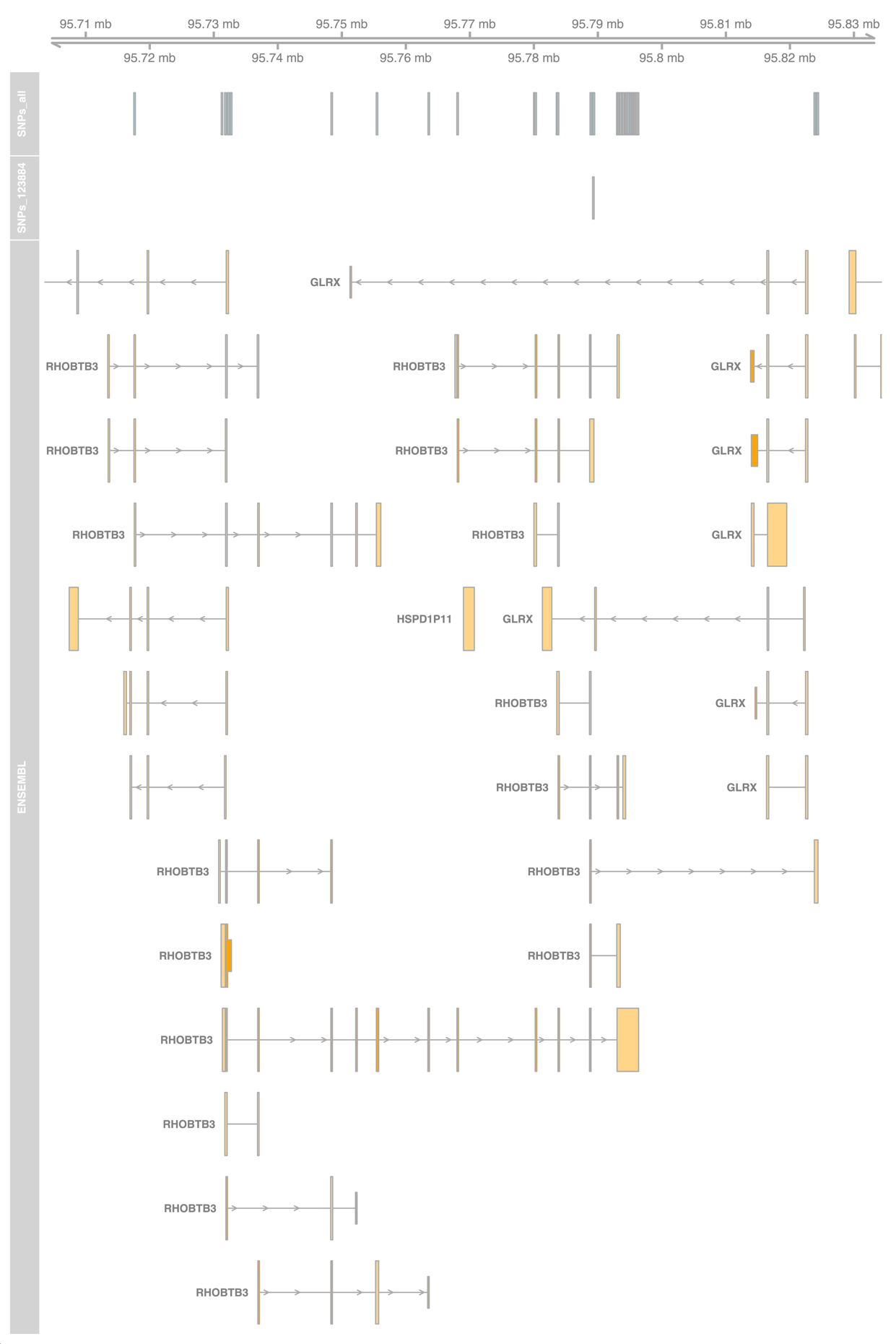


Supplementary Figure 2. SNP locations in the RHOBTB3 gene locus, with isoforms shown separately. The “SNPs_all” track shows all assayed heterozygous SNPs in this gene; the “SNPs_123884” track shows only the SNPs detected in the specified sample; and the “ENSEMBL” track displays the gene models.


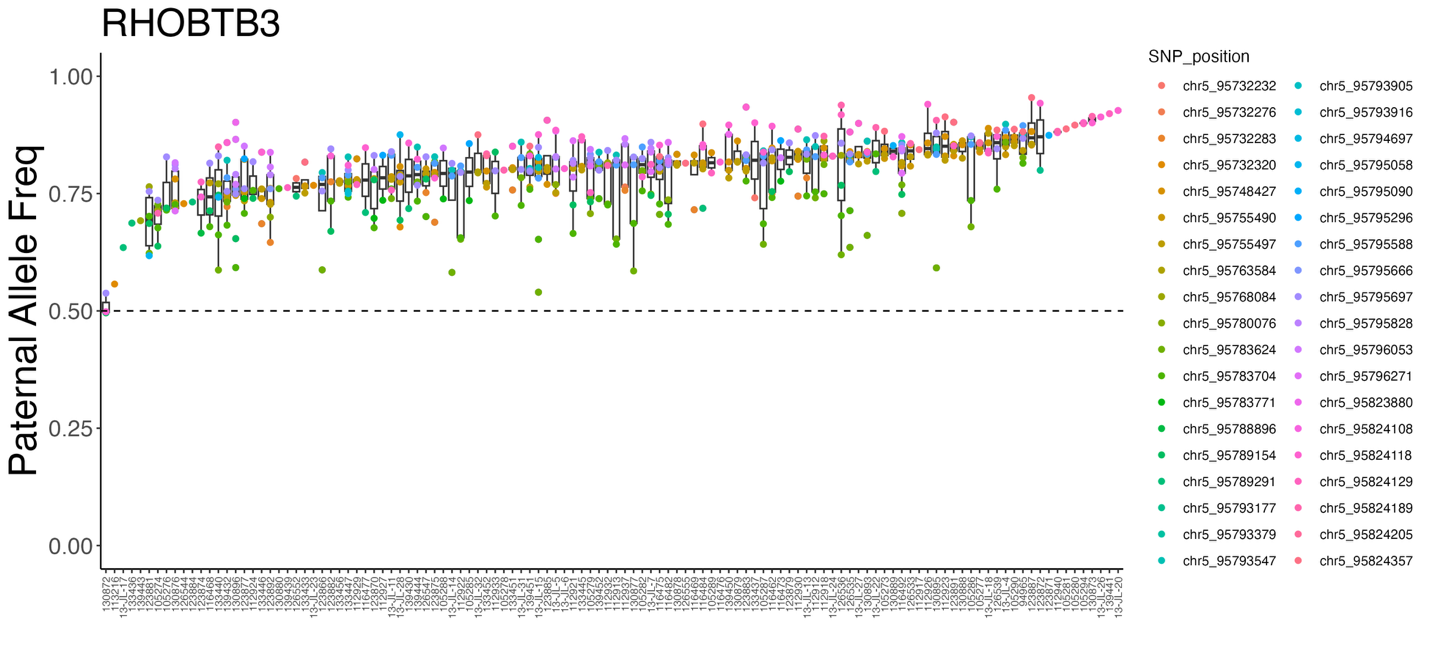


Supplementary Figure 3. Distribution of SNP-level paternal allele frequency across different samples in a gene, shown as a boxplot where each unique SNP position is indicated. The SNPs at the same location have the same color.

Supplementary Table 1. We compared the genes identified as imprinted by our PofO method with those reported by Hamada et al. (2016). Our PofO test generated a *po_z* score for each gene, and we classified genes with | *po_z* | > 3 as imprinted. In the Hamada dataset, allelic-specific expression (ASE) ratios and corrected binomial test p-values were reported in Supplementary Table 7, with p < 0.05 considered significant. Using this cutoff, we classified genes as either “Hamada_imprinted” or “Hamada_not_imprinted”. As our test data were derived from targeted RNA-Seq, 208 genes had both *po_z* scores and Hamada p-values available. A 2×2 contingency table from these genes is shown below. Fisher’s exact test yielded p = 8.9e-15 and an odds ratio of 12.7, indicating strong concordance between the two methods. Notably, our PofO model accounts for genetic effects, which may explain why many genes in the “Hamada_imprinted” group exhibited | *po_z* | scores below 3. In addition, the Hamada study primarily analyzed first- and second-trimester placentas, whereas our test data were from term placentas, which may also contribute to differences in imprinting classification between the two datasets.

|  | **Hamada_imprinted** | **Hamada_not_imprinted** |
| --- | --- | --- |
| **\|po_z\| > 3** | 71 | 11 |
| **\|po_z\| <= 3** | 42 | 84 |
